# Accessibility of diabetes education in the United States: barriers, policy implications, and the road ahead

**DOI:** 10.1093/haschl/qxae097

**Published:** 2024-08-21

**Authors:** Anna Tharakan, Eugenia McPeek Hinz, Emelia Zhu, Brad Denmeade, Jashalynn German, Wei Angel Huang, Amanda Brucker, Joanne Rinker, Chris Memering, Susan Spratt

**Affiliations:** Department of Undergraduate Studies, Duke University, Durham, NC 27710, United States; Margolis Center for Health Policy, Duke University, Durham, NC 27710, United States; Division of General Internal Medicine, Department of Medicine, Duke School of Medicine, Durham, NC 27710, United States; Duke Primary Care, Duke University Hospital, Durham, NC 27710, United States; Division of Endocrinology, Metabolism and Nutrition, Department of Medicine, Duke School of Medicine, Durham, NC 27710, United States; Division of Endocrinology, Metabolism and Nutrition, Department of Medicine, Duke School of Medicine, Durham, NC 27710, United States; Duke Department of Biostatistics and Bioinformatics, Duke University, Durham, NC 27710, United States; Duke Department of Biostatistics and Bioinformatics, Duke University, Durham, NC 27710, United States; Association of Diabetes Care & Education Specialists, Asheville, NC 28175, United States; North Carolina Diabetes Advisory Council, New Bern, NC 28560, United States; North Carolina Diabetes Advisory Council, New Bern, NC 28560, United States; Carolina East Medical Center, Carolina East Health System, New Bern, NC 28560, United States; Division of Endocrinology, Metabolism and Nutrition, Department of Medicine, Duke School of Medicine, Durham, NC 27710, United States; Population Health Management Office, Duke University Hospital, Durham, NC 27710, United States

**Keywords:** diabetes, diabetes education, diabetes self-management (DSMT), diabetes policy, prevention programs

## Abstract

Diabetes Self-Management Education and Support (DSMES) programs are an effective, yet underutilized, resource to improve health outcomes and behaviors for people with diabetes. We examined the attendance and referral rates for people with diabetes to DSMES classes at an academic medical center, noting a 10% referral rate and 37% completion rate for those referred. We identified barriers to DSMES care at patient, provider, and health system levels. Current technology platforms and training fail to prioritize referrals to diabetes education; providers and people with diabetes are often unfamiliar with program content and benefits. Scheduling mechanisms often delay or lose interested patients in receiving vital education. Existing Medicare reimbursement strategies limit expansion of DSMES programs, generating significant wait times and limit capabilities for Diabetes Care and Education Specialists. We identify potential policy solutions and recommend alterations to existing referral and scheduling systems to expand existing technology platforms for DSMES programs and shift reimbursement policies to individualize and better support care for persons with diabetes.

## Introduction

Diabetes mellitus is a costly chronic disease associated with significant morbidity and mortality affecting 38 million people in the United States.^1^ As the leading cause of blindness, kidney failure, and nontraumatic lower limb amputations,^2^ diabetes accounts for $503.4 billion of national health expenditure, reflecting 1 in 4 (25%) of all health care dollars.^3^ The largest contributors to high costs are prescription medications and inpatient care services, which can be reduced with individualized diabetes education.^3^

The Association of Diabetes Care and Education Specialists (ADCES) has identified 7 self-care behaviors for effective person-centered diabetes self-management: healthy coping, healthy eating, being active, taking medication, monitoring, reducing risk, and problem-solving. These behaviors are supported by the Diabetes Self-Management Education and Support (DSMES) program, which provides evidence-based training to empower people with diabetes to make informed self-management decisions.^4^

### Success with DSMES

The DSMES program is built upon proven strategies to improve health behaviors and outcomes.^5–7^ National data found that DSMES services reduce glycated hemoglobin (HbA1c) by 0.6%–1.0% overall in approximately 70% of participants, which has been our experience locally.^6^ This reduction in HbA1c is similar to most medication classes that treat diabetes, which highlights the power of DSMES and behavior change to manage diabetes.^8^ Individualized diabetes education has significantly improved the number of people with diabetes who reach their therapeutic targets and increased medication adherence and overall outcomes.^9^ The probability of health risk factors, such as emergency room visits, chronic complications, and strokes, significantly decreased for people with diabetes who attend DSMES.^9,10^ Diabetes education at Duke Primary Care Clinics (DPC) has seen consistent success for those who complete the entire program. In 2022, 880 people with diabetes (73.8%) who attended at least 1 class saw an average 1.5% reduction in their Alc (Susan Spratt, MD, Written, May 2024).

Participants who attend diabetes education have lower health care costs compared with their counterparts over a span of 3 years. With an average cost reduction of 12% within the education-receiving cohort, the savings in cost of care are substantial.^11^ While diabetes education is associated with increased use of primary and preventative services, there is an overall reduced health care cost due to the lower use of acute, inpatient hospital services.^11,12^ One study found that patients who receive diabetes education saved their health system an estimated $5287 per patient based on the estimated mean 3-year cost difference.^13^

### Barriers to entry for diabetes education

**Figure 1.**
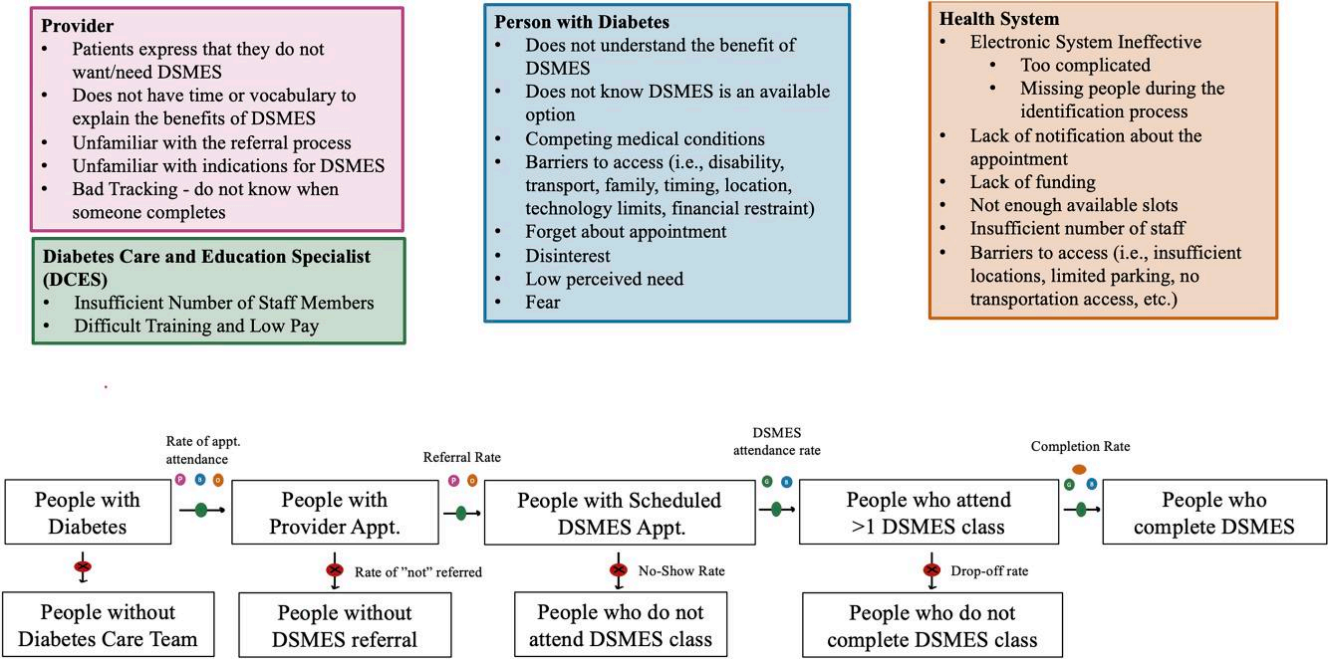
Process map of barriers to Diabetes Self-Management Education and Support Services (DSMES). The colors of the circles represent the specific barriers as alluded to in the corresponding colored box. Abbreviation: Appt., appointment.

Nationally, only 5%–7% of patients who are referred for DSMES through Medicare or private insurance receive it.^14^ We identified barriers to referral, enrollment, and completion of DSMES courses to improve attendance (Figure 1).

## Methods

This paper aims to identify the multiple factors that pose barriers to obtaining diabetes self-management education and support. We present a case study of the interaction of patients, providers, and the health system that affects access to diabetes education and policy solutions to promote the benefits of diabetes education programs nationwide. At a large academic health system and accountable care organization in the Southeast, we independently reviewed all people receiving diabetes care at our institution to determine the referral, scheduling, and completion patterns. Diabetes Care and Education Specialists (DCES) from the American Diabetes Association (ADA)–recognized programs in primary care and endocrinology departments were interviewed.

## Results

### A university health system case study: referral to and completion rate of DSMES programs

Of 56 638 patients with diabetes, only 10% (*n* = 5722) were referred and, of those, 37% (*n* = 2177) completed education. For patients with an HbA1c greater than 8.0, the referral rate was slightly higher (16.2%). We found that females (12%) were more likely to be referred to diabetes education compared with males (10%). Black patients (12%) were referred to diabetes education more often than White patients (8%). There were more Black patients with an HbA1c under 8% referred for diabetes education (*n* = 1503) than over 8% (*n* = 1069). White patients (29%) were also found to complete diabetes education more often than Black patients (25%) (Table 1).

**Table 1.** Patient demographics.

Patient characteristic	Total	Referred	Not referred
Age, mean (SD)	59.5	56.3	62.6
Sex, *n* (%)	56 638	5722 (10.1%)	50 916 (89.9%)
Male	26 269 (46.4%)	2455 (42.9%)	23 814 (46.8%)
Female	30 369 (53.6%)	3267 (57.1%)	27 102 (53.3%)
Race	56 638	5722	50 916
American Indian or Alaskan Native	298 (0.53%)	37 (0.64%)	261 (0.51%)
Asian	2495 (4.4%)	266 (4.6%)	2229 (4.4%)
Biracial	47 (0.083%)	15 (2.6%)	32 (0.063%)
Black or African American	21 320 (37.6%)	2572 (44.9%)	18 748 (36.8%)
Caucasian/White	29 828 (52.7%)	2516 (44.0%)	27 312 (53.6%)
Multi Racial	67 (1.2%)	8 (0.14%)	59 (0.12%)
Other or not Reported	2578 (4.6%)	308 (5.4%)	2270 (4.5%)
Ethnicity	56 638	5722	50 916
Hispanic/Latino	2339 (4.1%)	243 (4.2%)	2096 (4.1%)
Not Hispanic/Latino	52 295 (92.3%)	5184 (90.6%)	47 111 (92.5%)
Not Reported/Declined	2004 (3.5%)	295 (5.2%)	1709 (3.4%)

*Percentages listed in the row subsections are of the section that it is a part of

**Figure 2.**
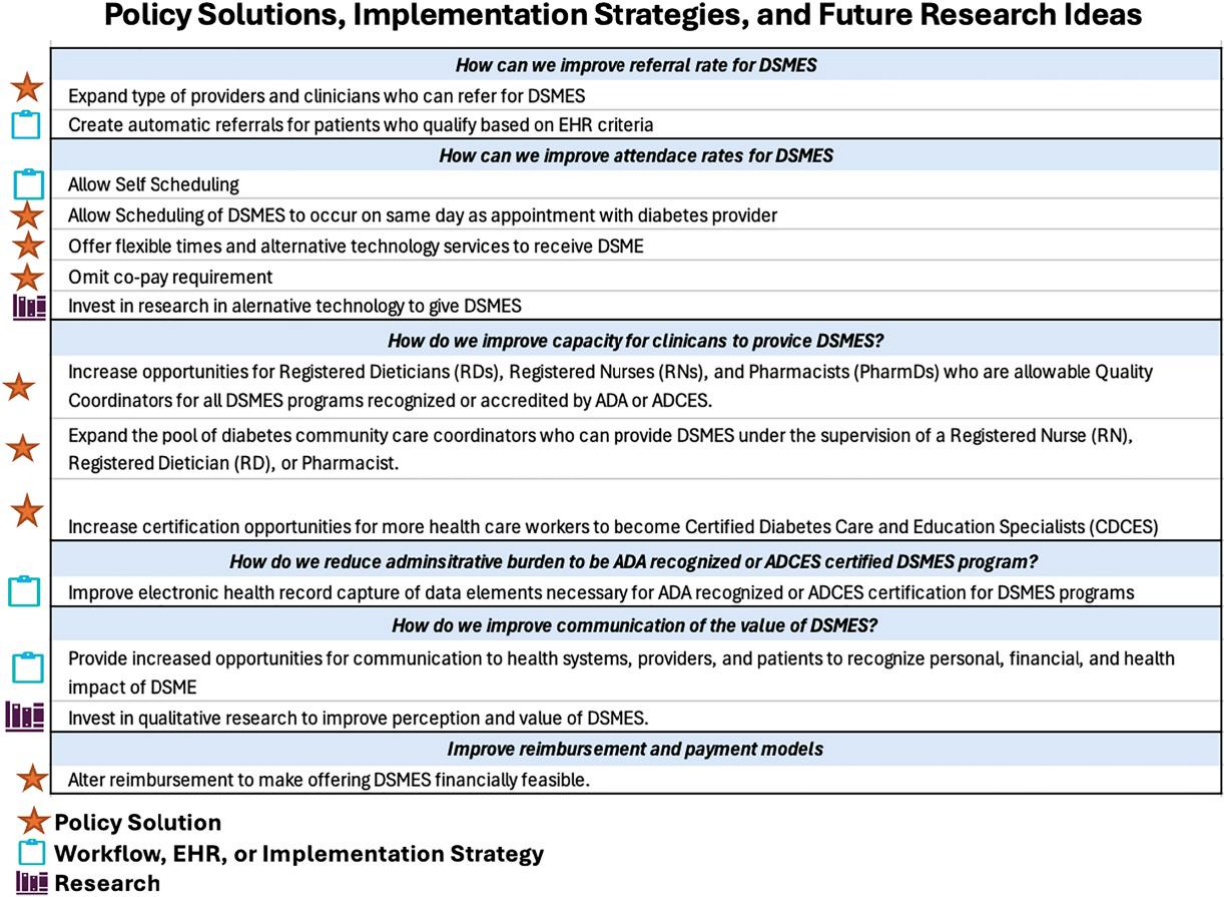
Policy solutions, implementation strategies, and future research ideas. Abbreviations: ADA, American Diabetes Association; ADCES, Association of Diabetes Care and Education Specialists; DSMES, Diabetes Self-Management Education and Support; EHR, electronic health record.

Consistent with the literature, patients who completed our DSMES program demonstrated improvement in glycemic control, with a lower average HbA1c compared with controls (Emelia Zhu, MPH, RD, LDN, CDCES, and Bran Denmeade, DNP, RN, CDCES, Written, August 2023). Diabetes Self-Management Education and Support is available at multiple primary care clinical and endocrinology sites across the institution. Our case study found that many people with diabetes and indications do not obtain DSMES services due to barriers in referrals, scheduling, and program capacity.

### Providers as gate keepers to referrals

For financial reimbursement, people with diabetes must be referred to DCES from the provider who treats their diabetes. Other clinical care providers, such as ophthalmologists, nephrologists, nurses, and inpatient providers, are not considered the diabetes provider and thus cannot refer. As such, a small subset of providers act as de facto gatekeepers for patients to attend DSMES classes. In our interviews with providers, they also expressed confusion over the referral process, were unaware of the curriculum and benefits, and reported that DSMES was not emphasized in their training as reasons for low referral rates.

### Health system

After the referral is placed, there remains a significant issue with scheduling. Of 3251 patients referred to the primary care DSMES program, 51.9% (*n* = 1687) of referrals were scheduled and only 858 (29%) of those completed.

After a referral is placed, patients receive 2 phone calls from a referral coordinator to schedule DSMES. If the appointment is not scheduled over the phone (ie, no answer, unsure of schedule or transportation), a letter is mailed to the patient with a reminder to call the coordinator. We identified that 98% of all the completed appointments were scheduled during the first 2 calls. Furthermore, only 68% of referrals where a letter was mailed were scheduled. Direct contact with patients, compared with indirect methods such as mailed letters, has higher success at scheduling.

The DCES voiced that, prior to COVID-19, the clinic allowed for front-desk scheduling immediately after the provider visit, a time when patient interest and knowledge are highest. This method was halted when the clinic pivoted to virtual visits during the COVID-19 pandemic. When return to work was possible, a hybrid model of in-person and virtual appointments occurred but the workflow of scheduling DSMES appointments directly after visits was not reinstated. Staff shortages compounded the problem, with further delays in scheduling.

### Perceived benefit of DSMES from people with diabetes

While the benefits of DSMES are widespread, they may not be obvious to people with diabetes. Diabetes education was described as “not worth the time,” “would [not] contribute anything,” or “did not need the additional help.”^15^ Given the fact that DSMES has been shown to lower HbA1c by 1%, the importance of DSMES for individual health is not well communicated. People with diabetes can face comorbidities or conditions that require them to attend multiple clinic visits; diabetes education may be seen as “just another appointment” where attendance is not prioritized. Furthermore, since DSMES classes do not provide novel medical information, such as test results or a physician visit, patients view the classes as information they could acquire “anywhere else,” or guidance that may not warrant a visit to the hospital or clinic.

Health-related social needs for transportation, technology limitations, financial restraints, or others further impair attendance. Although DSMES is a covered benefit, patients must pay a co-pay to attend. These obstacles hinder patients’ abilities to schedule and attend appointments.

### Capacity of DSMES programs

Even if all eligible patients were referred to and willing to attend DSMES, the current number of educators is insufficient to meet the demand. Nationwide there are 19 500 DCES available for the approximately 2 million new patients and the 12–18 million patients with uncontrolled diabetes. This translates to approximately 1 DCES per 1000 patients. The current DCES can only provide for 4 million patients per year or 20% of those with need. To meet the current demand, we calculate that an additional 35 000 DCES would be needed, nearly tripling the number of currently available educators. We propose a multipronged approach, extending the number of educators but also expanding the use of technology to meet the gap. We present our policy solutions, implementation strategies, and future research suggestions below (Figure 2).

## Policy solutions

### Pass the Expanding Access to DSMT Act

The Expanding Access to DSMT Act was introduced to Congress in 2018 to address barriers and extend benefits of diabetes self-management training (DSMT) beyond the first year of diagnosis. This act posits the following:

1. Expand the number of referring providers beyond the provider who manages the individual’s diabetes.
2. Extend the initial 10 hours of DSMT covered by Medicare beyond the first full year until used with an additional 6 hours annually.
3. Exclude DSMT from deductible requirements.
4. Allow DSMT to be serviced by community-based locations.
5. Launch a virtual DSMT demonstration project to test the effectiveness of virtual DSMT.^16^

This act, which has yet to be passed, will be monumental to support people with diabetes, as well as health systems that want to provide diabetes education. The Congressional Budget Office recently published its cost estimate for the bill, which found that, while bill implementation would increase health system utilization, it would also reduce acute-care service use, yielding a projected $0 implementation cost.^17^*We advocate for the immediate passage of the Expanding Access to DSMT Act to improve services and opportunities for providers, patients, and the health system.*

### Expand pool of clinicians who can refer to DSMES

To allow for a greater percentage of participants to attend DSMES, the current provider-referral process must be eliminated or greatly expanded. Medicare and Medicaid only reimburse clinicians for DSMES services if the patient is referred by their diabetes-treating provider (ie, primary care provider or endocrinologist). Persons with diabetes have an average of 1–2 visits per year with their diabetes-treating provider, who may not see them at the greatest time of need.^18^ Complications are often managed by inpatient facilities or specialists.^19^ Allowing more health care workers (ie, specialists, nurses, community health workers, pharmacists, dietitians, etc) to recommend DSMES services can improve access for patients when they need it the most. *We advocate that the Centers for Medicare and Medicaid Services (CMS) and the ADA expand the pool of clinicians who can refer to DSMES.*

### Shift scheduling processes to be completed immediately after the appointment

Specific standards for scheduling referrals will ensure the greatest attendance for DSMES appointments and largest scheduling rate post-referral. Currently, Medicare does not cover physician/provider visits and DSMES visits on the same day. For health systems that have co-located DSMES programs on site, this hurts retention and hinders relationships that could be fostered with DSMES team members. *Incorporate DSMES scheduling on the same day to improve education opportunities and retention.*

### Adjust certification requirements for new clinical sites to become certified to deliver and bill for DSMES

Programs must be recognized by the ADA or ADCES to be reimbursed. At present, there are 6 standards of care that are required to qualify for this recognition, which is a barrier for some programs that are not able to fulfill these requirements. Often, the programs that struggle to receive this certification are not directly affiliated with hospitals and are typically located in more rural areas. This certification stands as a significant barrier for access to crucial services for a significant at-risk population. *Adjust the standards of care to accommodate more programs to increase the number of available clinics and services to patients, specifically those in rural areas.*

### Train and hire a larger workforce to meet demand and provide physical and technological infrastructure support

Access to DCES, registered dietitians, nurses, and pharmacists will require training and hiring a larger workforce, along with physical and technological infrastructure support to meet the demand. The workforce can be expanded through 2 avenues:

1. Increase opportunities for registered dietitians (RDs), registered nurses (RNs), and pharmacists (PharmDs) who are allowable quality coordinators for all DSMES programs recognized or accredited by ADA or ADCES. Once a program becomes ADA certified, there must be at least 1 RN, RD, or PharmD that supervises all other staff, known as the quality coordinator. All other staff have no limitations on their qualifications (ie, can be a community health worker [CHW]) but require 15 hours of continued education in diabetes annually. Increased opportunities and incentives for quality coordinators to facilitate and supervise DSMES programs will increase the available programs and support available for patients that request these services.
2. Expand the pool of diabetes community care coordinators who can provide DSMES under the supervision of a quality coordinator. To improve capabilities and support of DSMES programs accredited by ADA or ADCES, there must be increased opportunities for diabetes community care coordinators. One proposal to improve training pathways is for CHWs to become diabetes community care coordinators, which will increase accessibility and workforce for diabetes education classes. Community health workers are complementary health care workers who interact with people with diabetes. They can provide education, guidance, and support to help individuals build self-management skills.^20^ Due to their work in specific community-based settings, they understand the challenges of care that people with diabetes may face, and therefore can help with care-management strategies that can be lifestyle specific. They can provide DSMES under supervision. Community health workers may be able to host diabetes education classes, provide culturally sensitive nutritional information, and help adopt lifestyle changes that the patient may require. *Increase avenues to training and certification for existing CHWs to allow for expansion of the workforce and provide more community-based alternatives.*

### Increase certification opportunities for more health care workers to become Certified Diabetes Care and Education Specialists through improved certification timeline and requirements

Certification to become a Certified Diabetes Care and Education Specialist (CDCES) is time consuming, unpaid, and requires work on the learner to find an appropriate training site. Current training is considered extremely rigorous and time consuming, which deters new health care workers to consider the pathway.

Requirements include an extensive course, along with 1000 hours of diabetes education experience. The process to complete this certification could come earlier, during training, or made more accessible outside the workday, after hours, offered online, or with a shortened clinical time requirement if that participant works in a diabetes clinic after completion of the program. *Support funding methods for training sites and participants for accelerated credentialling pathways.*

### Alter reimbursement requirements so that diabetes education clinics can be financially supported

While the benefits of diabetes education universally exist, they are not fully understood and advertised. Health systems and administrators do not view diabetes education as a crucial part of diabetes care but rather as an extra resource that many patients choose not to utilize. Coverage for DSMES has typically been reimbursed by the CMS, which has encouraged other insurance carriers to do the same. Coverage for specific evaluation (continuous glucose monitor/insulin pump), education (diabetes device or diabetes technology), or behavioral interventions, which may take shorter time periods, should be established. *For any of these policy changes to occur, there must be significant changes made to reimbursement.*

### Self-scheduling workflows for DSMES in the electronic health record tool and implementation strategies

We propose that any person with diabetes who qualifies should have the ability to access education whenever they would like. Standardizing this approach could remove the burden from primary care providers and allow task-sharing across disciplines.^21^ Diabetes education referrals can be expanded to the electronic health record (EHR) platforms, where patients can schedule a DSMES appointment based on indications identified via their EHR (ie, HbA1c values >7.0%, etc). Electronic health records can be equipped to include automatic standing orders for all people with diabetes at any of the 4 critical referral times: at diagnosis, annually/when no treatment targets are not met, when complicating factors develop, and when transitions in life and care occur.^22^ Automating referrals within the EHR creates an opt-out rather than an opt-in workflow. This practice has been shown to be effective within other health care referral processes, such as with mammograms.^20^ Additionally, after program completion, all communication about the success of the program and improved outcomes of the participant should be shared with the referring provider. *Self-scheduling workflows should be made available for DSMES services.*

### Communication of indications, content, and benefits for DSMES

Duke Primary Care Clinics, a consortium of 37 practices and 9 urgent care clinics, have diabetes education providers located at 4 sites. They presented diabetes education at DPC provider meetings, which resulted in a 30% increase in referrals. This is attributed to providers’ improved ability to understand the program and explain the process to people with diabetes. However, large time demands limit provider attendance. Programs should integrate similar sessions into routine practice or existing meeting requirements, which would minimize extraneous time commitments and allow providers and people with diabetes to reap its benefits. *Provide increased opportunities for communication to health systems, providers, and patients to recognize the personal, financial, and health impact of diabetes education.*

### Improve reporting through discrete data capture of DSMES goals and outcomes within electronic health records.

To achieve certification, programs often struggle with reporting certain required elements, including patient goals, percentage of patients that completed goal, HbA1c improvement displayed by patients with completed education, demographics, etc. The local academic university implemented a report within the EHR system, which allows DCES and program managers to assess programs without manual chart review. Certified Diabetes Care and Education Specialist RNs and RDs use note templates that capture discrete data that accreditation requires. *Use of discrete data captured in the EHR improves efficiency to obtain or maintain ADA or ADCES certification for DSMES programs.*

### Research alternative technology solutions and qualitative research

#### Introduce and research alternative technology solutions to expand diabetes education services

For patients unable to utilize DSMES services, technology solutions should be proposed as an alternative. Health education companies, such as Healthwise, offer health education videos and classes that participants can utilize as a supplemental source of information. Diabetes education can be offered through this virtual form as well. These courses can often be personalized to match the stage of diabetes education that the patient requires at that time. For example, Healthwise outlines 5 stages of change, each with their own stage-specific content that allows patients’ educational material to be uniquely curated to match their needs.^23^ For patients who do not have the resources to attend DSMES classes, want supplemental education, or cannot obtain a referral, they can use services such as Healthwise to acquire some diabetes education.

There are several online resources, such as classes, websites, and telephone apps, that teach about diabetes and provide coaching and medication assistance.^24^ Apps such as CDCES Coach App and MHealth MyDiabetes provide expert advice, structured education, nutritional guidance, and exercise programs, with options.^1^ Several universities and health systems offer online courses to people with diabetes with focuses on nutrition, exercise, medications, and self-management strategies. This is consistent with in-person DSMES curriculum that covers the ADCES7: Healthy eating, being active, monitoring health, taking medication, problem-solving, healthy coping, and reducing risky behaviors. However, many of these class offerings are limited and levels of personalization differ.

Coverage and alternatives to in-person DSMES classes can expand availability for participants, as well as provide feasible alternatives to patients who have language or accessibility barriers. Technology can help expand DSMES to meet the needs of patients who require more sensitivity when it comes to accommodation for different lifestyles. *Reliable technology options that are properly advertised and supported can expand opportunities for patients unable to utilize currently offered DSMES services.*

#### Funding qualitative research

There are many gaps in the knowledge about low referral and attendance rates for DSMES. Formal qualitative research to understand why providers do not refer, why patients do not show to appointments or complete their referral, and why current communication about diabetes education benefits may not be the most effective is imperative before implementation of large-scale policies to target these barriers. Furthermore, research must address feasibility studies on different DSMES methods, specifically to understand how to reach more patients. Funds for cost-analysis studies will help health systems realize return on the investment in implementation of DSMES. This research can help create a larger public ad campaign designed for the elimination of myths regarding diabetes education and improve attendance. *Investment in research on how to improve the perception and value of DSMES will support policies that encourage diabetes self-management education.*

## Limitations and future studies

This case study had a few limitations that hinder the generalizability of the results. First, while many health care systems have DSMES programs, these results are limited to 1 academic health system, and therefore, may not be entirely representative of the nation; but overall, the barriers nationally are similar. Our study did not explore the feasibility of the policy options. Future studies are required to understand the entire reach of DSMES nationally. The proposed policy solutions need to be tested and explored to understand their feasibility.

More needs to be done to understand the return on investment on a health-system level. Through this, funding can be reallocated to better support the growth of sites that deliver DSMES, specifically through better scheduling systems and more staff. Health systems and patients will benefit from increased expenditure for diabetes education.

## Conclusion

Diabetes self-management education is an essential resource for diabetes care, but its low utilization limits the impact it can have on persons with diabetes. With its individualized approach, DSMES clinics have been shown to create significant improvement in glycemic management for people with diabetes and reduce health care costs for health systems. Changes are required within the referral system, scheduling process, reimbursement, workforce capacity, and provider-patient education to increase attendance for diabetes education.

## Supplementary Material

qxae097_Supplementary_Data
